# Prevalence and impact of parental alienating behaviors (PABs) in adults aged 18–25 in the United Kingdom

**DOI:** 10.3389/fpubh.2026.1803173

**Published:** 2026-04-23

**Authors:** Benjamin A. Hine, Jennifer J. Harman, Sadie Leder-Elder, Elizabeth A. Bates

**Affiliations:** 1 University of West London, Ealing, United Kingdom; 2 Colorado State University, Fort Collins, CO, United States; 3 High Point University, High Point, NC, United States; 4 University of Cumbria, Carlisle, United Kingdom

**Keywords:** depression, family violence, mental health, parental alienating behaviors, parental alienation, PTSD

## Abstract

**Introduction:**

Following recent research in the United Kingdom (UK) on the prevalence of parental alienating behaviors (PABs) as reported by separated parents, this study presents the first nationally representative investigation of the prevalence and impacts of PABs as reported by young adults in the United Kingdom.

**Methods:**

A sample of 1,004 participants aged 18–25 completed an online survey assessing exposure to 30 established PABs, parental acceptance and rejection, and mental health outcomes, including post-traumatic stress, depression, and suicidality.

**Results:**

Results revealed that 98.3% of participants reported experiencing at least one PAB from a parent during childhood, with over half reporting exposure to ten or more behaviors. Approximately one-quarter experienced twenty or more behaviors. Males reported significantly higher exposure to PABs than females, though no other demographic differences were found. Exposure to PABs was significantly correlated with increased parental rejection and decreased parental acceptance, as well as elevated symptoms of PTSD, depression, and suicidal ideation.

**Discussion:**

These findings provide robust evidence that PABs are both widespread and psychologically harmful, underscoring their recognition as a form of family violence with long-term consequences. Implications include the need for comprehensive legal frameworks, enhanced clinical training, and public awareness initiatives to address and mitigate the effects of PABs on children and families. Future longitudinal research is needed to better understand trajectories of resilience and vulnerability among affected individuals.

## Introduction

Parental alienating behaviors (PABs) are actions taken by one parent that contribute to the child's alignment with the perpetrating parent and their rejection of the targeted parent (1). These behaviors can result in what is known as “parental alienation” (PA), defined as “one type of contact refusal when a child— typically whose parents are engaged in a high-conflict separation or divorce—allies strongly with one parent and resists and rejects contact and/or a relationship (i.e., contact refusal) with the other parent without legitimate justification” [(2), p. 5]. In other words, PA refers to the actions and attitudes manifested by the child when a psychologically and coercively controlling abusive dynamic exists in the family system, and the child(ren) are utilized as part of this dynamic. Indeed, recent research has increasingly and appropriately positioned PABs as a form of family violence, linking them to coercive control, psychological abuse, and child abuse (3, 4). Moreover, testimonies from alienated parents themselves (5) and case review evidence from studies in the US and Canada reveal a strong overlap between PABs and other forms of intimate partner violence (6, 7) and that PABs only need to be perpetrated once to be considered “abusive” (8).

Research on PA has expanded rapidly over the last decade (9); Hine, (10) and there is now a robust evidence base relating to many aspects of PA, including how it develops (i.e., the identification of PABs), how it is measured, how it differs from other forms of contact refusal, and pathways to intervention (9). This is despite some scholars questioning the scientific foundations of PA (11), with the emergent evidence suggesting this criticism is unfounded. Research has also outlined the extensive impact PA has on alienated parents (5), children (12), and other family members (13), as well as its complex application in legal disputes on custody and child contact (7, 14–16). Moreover, several studies have highlighted that alienating parents often purposefully misuse legal and social services to further isolate the targeted parent and damage their parent-child bond, thus aligning PA with legal and administrative abuse (17–21). Taken together, these findings highlight the severity of PA and underscore the need for effective legal and social interventions.

Until recently, assessment of the prevalence of PABs was limited to the United States and Canada (22, 23). However, a recent study has begun to investigate the prevalence and impact of PABs in the UK (24). A survey of over 1,000 separated or divorced parents revealed that, in line with previous studies, 39.2% of respondents acknowledged experiencing PABs when asked directly. This figure rose to 59.1% when specific behaviors were measured, thus highlighting the widespread nature of PABs. This study also demonstrated a clear relationship between experiences of PABs and various mental health measures, including PTSD, depression, and suicidal thoughts (though the directionality of this relationship is still unclear). Additionally, a correlation was found between PABs and other forms of domestic abuse reported by participants, further supporting the positioning of PABs as a form of intimate partner violence. Hine et al. (24) concluded that, along with further research in the UK and worldwide, a dual strategy is necessary: First, enhancing mental health support; and second, raise public awareness through comprehensive campaigns. Crucially, it was argued that these findings provide the critical impetus for future policymaking and intervention strategies aimed at mitigating the impact of PABs on affected families due to their prevalence and negative consequences to families.

The prevalence and impact of alienating behaviors, as experienced and reported by children, is even less understood. Research undertaken by Italian researchers has provided preliminary evidence that PABs are widely experienced by children and that there are long-lasting effects into adulthood (specifically on mental health outcomes) (25–28). However, this data was gathered from convenience samples rather than a sample reflecting the general population and is limited to one country. Marsden and colleagues (29) found that a new PA screening item added to the original Adverse Childhood Experiences (ACEs) measure explained significantly more variance in negative childhood outcomes such as depression and PTSD than the original measure, and the item correlated highly with other household dysfunction items on the scale. Conducting further investigation of PAB prevalence in different countries and using representative samples would likely expand and support the results above in demonstrating both the high prevalence of PABs experienced by children and their impact on children when they are experienced and well into adulthood (12).

The present study, therefore, utilized measures like those used in Hine et al. (24) to conduct the first-ever UK study on the prevalence of PABs in children, as reported by those children as young adults. Using a specialist research panel service, a representative sample of over 1,000 adults aged between 18 and 25 in the UK were surveyed and asked questions used in previous prevalence research on this topic (22, 23, 27), to create directly comparable UK data. This method provided the only and most comprehensive assessment of the scale of this issue within the UK to date.

## Methods

### Sample

Participants were 1,004 residents of the UK, all aged between 18 and 25 years old. This was the only inclusion criteria. The average age for the sample was 21.24 years (*SD* = 2.04). Two hundred and forty-four (24.3%) of the sample identified as male, with 804 (80.1%) identifying as heterosexual (11.1% Bisexual, 4.7% Lesbian/Gay, 4.2% Other). Most of the sample identified as White (69.8%), with 14.9% identifying as Asian/Asian British, 8.6% as Black/Black British, 5.5% as Mixed or Multiple, and 1.2% as Other. The most common household income bracket was £20,001–£30,000, followed by £30,001–£40,000, £10,001–£20,000, and then £40,001–£50,000 (55.7% of the sample). About 1/5 of the sample (20.8%) had secondary school qualifications as their highest qualification, with a further 43.5% and 25.1% having A-level/equivalent and bachelor's degrees as theirs, respectively (89.4% of the sample). Half of the sample (50.6%) were single, with the next highest categories being in a committed relationship (not cohabiting or married) (18.8%), married or in a civil partnership (18.5%), and cohabiting (10.2%). Based on available national figures for sexual orientation, and ethnicity distributions, this sample can be classified as broadly representative of the UK population, although there were proportionally fewer men than women, and slightly higher percentages of sexual and ethnic minorities than the general population (30).

### Measures

#### Positive co-parenting behaviors

Participants reported on the extent they recalled their primary caregivers engaging in nine different positive co-parenting behaviors (e.g., *my parents supported each other in disciplining me*) using a 5-point semantic differential scale (*strongly disagree* to *strongly agree)*. This scale was reliable (α = 0.89), and the items were averaged together to produce an overall score.

#### Parental acceptance

The Parental Acceptance-Rejection Questionnaire (PARQ) is a self-report questionnaire designed to assess children's current perceptions and adults' retrospective remembrances of the degree to which they experienced parental (maternal and paternal) acceptance or rejection in childhood (31). The measure consists of four scales: (1) warmth and affection (or coldness and lack of affection, when reverse scored), (2) hostility and aggression, (3) indifference and neglect, and (4) undifferentiated rejection. Undifferentiated rejection refers to individuals' feelings that the parent does not really love them, want them, appreciate them, or care about them in some other way without necessarily having any objective indicator that the parent is cold, aggressive, or neglecting. Collectively, the four scales constitute an overall measure of perceived or remembered parental acceptance-rejection in childhood. This scale was administered twice to participants, once for mothers and once for fathers, with Cronbach's alphas of 0.85 for both, demonstrating excellent reliability.

#### Parental rejection

We utilized five items from the Baker Alienation Questionnaire [BAQ: (32)] to measure parental rejection to further validate participants' childhood relationships and alignment with each parent. These were: “Do you think [Mother's Name] was/is a good caregiver to you?,” “How many good memories do you have of being with [Mother's Name]?,” “How much do you think [Mother's Name] could be/could have been a better caregiver?,” and “Do/did you enjoy spending time with [Mother's Name]?,” and “How angry or unhappy with [Mother's Name] are you right now?.” Participants were asked these questions twice – once for mothers and once for fathers. When asking about mothers, Cronbach's alpha was 0.79, and for fathers, the value was 0.85, suggesting high reliability.

#### Alienating behaviors

Participants rated 30 different behaviors that have been identified in the research literature as PABs (33). These are listed in Appendix A, and included items such as “My mother made me feel guilty if I enjoyed time with my father” and “My mother encouraged me to spend less time with my father” (See Appendix A for the full list of these behaviors). Reactance is a potential concern in surveying individuals who were alienated from a parent, as the alignment with the preferred parent can influence perceptions that they are “perfect” and are justified in their behaviors. For example, if an alienated individual is asked whether their mother or father spoke badly about their other parent, it is unlikely they will report the parent did this because they believe they were only telling the “truth” about them. The items used in this study were created for another research study (Harman et al., under review)^1^ to minimize this potential reactance in respondents. The 30 items used in this study were developed based on a broad array of different behaviors (e.g., loyalty inducing) rather than just parental denigration, and two positive behaviors were included to minimize response bias, and these were reverse-scored. Participants rated each behavior for their mother and father separately, and the reliability of the scales was high (α = 0.92 and 0.93, respectively).

#### Mental health

We assessed post-traumatic stress symptoms using a shortened version of the PTSD Checklist (34). Seven problems were selected from the original item list of 17 due to concerns about survey fatigue, and respondents were asked to indicate how much each of the seven problems had bothered them in the last month (using a 5-point scale with not at all and extremely serving as anchors). The items formed a reliable scale (α = 0.92), and they were averaged together.

We also administered a 20-item depression screening tool published by the Center for Epidemiological Studies (35) to assess depressive symptoms. Respondents rated how often in the last week they have felt certain ways (e.g., I was bothered by things that usually don't bother me), and respondents answered with rarely or none of the time (less than a day), some or a little of the time (1–2 days), occasionally or a moderate amount of time (3–4 days), and most or all of the time (5–7 days). The scoring of the measure is a summed score across the 20 items (4 of which are reverse scored) so that the range of scores is between 0 and 60, with higher scores indicating greater levels of depression. The reliability of this scale was high (α = 0.86).

We assessed suicidality by asking respondents whether and how often they have thought about suicide in the last year [never, rarely (1 time), sometimes (2 times), often (3–4 times) and very often (5 or more times)]. For those participants who did not answer “never” for whether they have thought about suicide in the last year, we then asked whether their thinking about suicide in the last year was related to conflict around their child custody situation with their ex (using a 5-point scale with *strongly disagree* and *strongly agree* as endpoints). Finally, we asked participants who had contemplated suicide in the last year whether they knew anyone who committed suicide due to child custody issues with their ex-partners (Yes, No, I don't know/Don't care to say).

### Procedure

The study was conducted through an online, mixed-methods survey, facilitated by Atomik Research—an independent creative market research agency accredited with Market Research Society (MRS)-certificated researchers and abides by the MRS code. It was carried out over two weeks from the 21st of February to the 3rd of March 2024. Participants for this study were recruited from an online consumer panel known as the ‘Power of Opinions'. They were chosen based on specific criteria: being aged 18–24 years old, residing in the UK, and being willing to consent to the study requirements.

The sample was drawn using a probability sampling methodology, and a total of 1,004 respondents participated in the survey. It is important to note that there were no hard quotas, and the socio-demographic composition was a natural fallout within this subgroup of the general population. However, as previously mentioned, based on available national figures for demographic distributions, this sample can be classified as largely representative of the UK population, with an over-representation of women and those from ethnic minorities.

The sample was drawn using a probability sampling approach, with the initial survey distributed to a nationally representative (nat-rep) general population sample of 10,000 respondents based on Office for National Statistics (ONS) data for gender, age, and regional distribution in England and Wales. From this broader sample, 1,005 respondents who met the criteria of being 18–25 years old participated in the study. This approach aimed to approximate a nationally representative distribution across key demographic variables.

The data collection process entailed the use of self-report questionnaires administered online. These questionnaires were divided into multiple sections, such as qualification, social demographic, and sections related to harmful and abusive behaviors. Upon completion of the study, qualified respondents were rewarded with a £5 incentive for their participation.

### Ethics

Ethical approval for this study was obtained from the Institutional Review Board (IRB) at a UK University prior to data collection. The study was reviewed and approved as a low-risk research project involving adult participants.

All procedures were conducted in accordance with institutional ethical guidelines and the principles of the Declaration of Helsinki. Participants were provided with information about the purpose of the study and gave informed consent prior to participation. Participation was voluntary and respondents were free to withdraw at any time. No personally identifiable information was collected, and all responses were recorded anonymously. Data were handled in accordance with UK data protection regulations, and strict measures were taken to ensure the confidentiality and anonymity of participants' responses.

### Statistics

All analyses were conducted using SPSS (Version 24). Descriptive statistics were first calculated to examine the prevalence and distribution of parental alienating behaviors (PABs) and related variables. Prior to conducting inferential analyses, the distribution of variables was assessed through inspection of skewness and kurtosis statistics and visual inspection of histograms. These indicated that the variables were within acceptable limits for parametric analysis.

Independent samples *t*-tests were conducted to examine gender differences in exposure to PABs and mental health outcomes. Bivariate Pearson correlations were used to assess relationships between PAB exposure, parental acceptance and rejection, and mental health variables. Statistical significance was set at *p* < 0.05.

## Results

### Contextual Information

Most of the sample (84%) reported having two parents or caregivers during their childhood (16% had a single parent/caregiver). Most of the sample reported having two parents present in the household for most of their childhood (71.5%), while 18.2% reported having one parent only, A small proportion of the sample (6.3%) had one parent and a stepparent, and 3.1% reported having one parent and another relative or family friend (0.9% other) as their caregivers. When asked about the family composition they experienced most recently, these figures were 65.1%, 22.7%, 8.8%, and 2.9% respectively, suggesting that as childhood progressed, some children transitioned from two to one-parent households. When reporting on the parental relationship, 58.5% of the sample reported their parents as being married for their whole childhood. 15.2% reported a divorce, 12.1% reported a separation, 2.7% reported a widowing, and 10.3% said they were never married (1.3% other). Of those whose parents were divorced or separated. See Table 1 for figures on whom participants lived with during childhood.

**Table 1:** Child contact arrangements for children whose parents were separated or divorced in childhood.

Child contact arrangement	Freq	% Sub sample	% Whole sample
It was fairly equal	39	14.2	3.9
I lived primarily with my mother and had some parenting time with my father	136	49.6	13.5
I lived primarily with my father and had some parenting time with my mother	19	6.9	1.9
I lived entirely with my mother and never or almost never had parenting time with my father	68	24.8	6.8
I lived entirely with my father and never or almost never had parenting time with my mother	7	2.6	0.7
Other	5	1.9	0.5

Most participants (93.8%) reported having a biological mother growing up, with 2.5% reporting an adopted mother, and 3.7% reporting no mother figure. Most participants (86.7%) reported having a biological father growing up, with 3.7% reporting an adopted father, and 9.7% reporting no father figure (noticeably higher than the same figure for mothers).

### Prevalence of PABs

Participants reported on the frequency of PABs for both parental figures. The mean score for reports of mothers' alienating behaviors was 2.49 (*SD* = 0.72), which equates to between rarely (2) and sometimes (3) on the scale. This outcome was similar for ratings of fathers (*M* = 2.42, *SD* = 0.76). Interestingly, scores on the positive co-parenting behaviors scale were not correlated to reports of PABs (the expectation being that this would be a negative relationship).

When looking at the sum of individual responses for mothers' behaviors, 90.7% of participants answered “often” or “most of the time” to at least one item. Almost a quarter (22.1%) of the sample answered in this way to at least 10 items, and 4.2% answered this to at least 20 of the 30 items. For fathers' behaviors, 87.4% of participants answered “often” or “most of the time” to at least one item. About one-third (21.4%) of the sample answered in this way to at least 10 items, and 3.2% answered this to at least 20 of the 30 items.

When including “sometimes” responses (3 on the scale) as a “yes” value, 98.3% of participants answered affirmatively to at least one item. Over half (58.5%) of the sample answered in this way to at least 10 items, and 25.9% answered this to at least 20 of the 30 items. For fathers' behaviors, 95.7% of participants answered “often” or “most of the time” to at least one item. Over half (53.1%) of the sample answered in this way to at least 10 items, and 25.1% answered this to at least 20 of the 30 items.

Men in the sample reported having experienced more alienating behaviors from mothers (*M* = 2.74, *SD* = 0.85) than women (*M* = 2.42, *SD* = 0.66), *t*_(944)_ = 5.89, *p* < 0.001. Men also reported having experienced more alienating behaviors from fathers (*M* = 2.67, *SD* = 0.89) compared to women (*M* = 2.35, *SD* = 0.69), *t*_(884)_ = 5.38, *p* < 0.001.

No other demographic characteristics were statistically significant from each other *(p*s > 0.05). Mothers' alienating behaviors and fathers' alienating behaviors were also highly correlated with one another, *r*(894) = 0.783, *p* < 0.001.

### Feelings toward parents

On reporting feelings about their mother (where one was present), 85.8% reported that the mother figure was somewhat to completely good to them, 85.7% reported some to very many good memories of them, 54.7% reported that they could “not at all” or “have been a little bit” a better caregiver, and 88.9% reported somewhat to very much enjoying spending time with them. Reporting on the present, 69% stated they were “not at all” or “a little bit” angry or upset with their mother now, and 68.6% reported that they had “not at all” or “a little bit” done something to hurt their mother. Just over half (52.2%) reported that, in disagreements, sometimes the mother and sometimes the other caregiver were right, followed by 43% reporting that always the mother was right. Most participants reported that feelings about their mother came only from them (61.9%), with 35.1% reporting partly from them and partly from others (3% only others).

On reporting feelings about their father (where one was present), 77% reported that the father figure was somewhat to completely good to them, 73.7% reported some to very many good memories of them, 45% reported that they could “not at all” or “have been a little bit” a better caregiver, and 78.1% reported somewhat to very much spending time with them. Reporting on the present, 61.7% stated they were “not at all” or “a little bit” angry or upset with their father now, and 68.7% reported that they had “not at all” or “a little bit” done something to hurt their father. Just over half (56%) reported that, in disagreements, sometimes the father and sometimes the other caregiver were right, followed by 24.3% reporting that always the father was right. Most participants reported that feelings about their father came only from them (53.9%), with 40.2% reporting partly from them and partly from others (5.8% only others).

### PABs and acceptance/rejection

Like other studies that have sought to understand the context and potential outcomes of PABs, several bivariate correlations were conducted to examine the relationship between mothers' and fathers' PABs and participants' reports of parental acceptance and rejection (see Table 2). As shown, both mothers' and fathers' PABs were significantly negatively correlated with parental acceptance (as measured by the PARQ). PABs were also negatively correlated with scores on the BAQ items assessing parental caregiving. Because the BAQ items were scored in a positive direction (higher scores reflecting more positive caregiving and lower perceived rejection), these negative correlations indicate that higher exposure to PABs was associated with lower caregiving scores and therefore greater perceived parental rejection. Taken together, these findings suggest that as alienating behaviors increased, perceptions of parental rejection increased while parental acceptance decreased.

**Table 2:** Correlations between mothers' and fathers' alienation and participants' acceptance/rejection (male participants above diagonal, female below).

Measure	Mother alienation	Father alienation	Mother acceptance	Father acceptance	Mother rejection	Father rejection
Mother alienation	–	0.860^*^	−0.539^*^	−0.668^*^	−0.358^*^	−0.288^*^
Father alienation	0.735^*^	–	−0.507^*^	−0.613^*^	−0.402^*^	−0.251^*^
Mother acceptance	−0.441^*^	−0.419^*^	–	0.647^*^	0.372^*^	0.331^*^
Father acceptance	−0.482^*^	−0.530^*^	0.577^*^	–	0.373^*^	0.286^*^
Mother rejection	−0.373^*^	−0.395^*^	0.456^*^	0.332^*^	–	0.454^*^
Father rejection	−0.367^*^	−0.245^*^	0.302^*^	0.281^*^	0.347^*^	–

–^*^ = *p* < 0.01.

### Mental health outcomes

Women in the sample had significantly higher PTSD and depression scores than men, as well as higher scores on three out of four suicide measures (see Table 3). For the whole sample, there was a positive correlation between the mothers' [*r*_(965)_ = 0.35, *p* < 0.001], and between fathers' PABs and PTSD scores [*r*__(_905)_ = 0.32, *p* < 0.001]. There was also a positive correlation between mothers' [*r*_(965)_ =.36, *p* < 0.001] and fathers' PABs and child depression scores [*r*_(905)_ =.325, *p* < 0.001]. Moreover, there was a positive correlation between mothers' PABs and all suicide-related questions (*p* < 0.01), and between fathers' PABs and all but one suicide-related question (“Have you thought about killing yourself in the last year?”), with all of these effects holding for male and female participants.

**Table 3:** Mean scores for mental health measures and statistical differences between male and female participants.

Measure	Male participants score (SD)	Female participants score (SD)	*T*-value	df
PTSD	2.47 (1.07)	2.63 (1.10)	2.05^*^	981
Depression	2.16 (0.56)	2.23 (0.51)	2.08^*^	981
Suicide thought or attempt (ever)	1.99 (1.44)	2.38 (1.60)	3.56^*^	458.7
Told someone going to commit suicide	1.55 (1.02)	1.74 (1.21)	2.38^*^	487.7
Likelihood of suicide someday	2.18 (1.69)	2.31 (1.56)	1.03	981
How often thought of suicide in past year	1.72 (1.18)	2.06 (1.33)	3.74^*^	461.8

–^*^ = *p* < 0.01.

## Discussion

To further understand the complex dynamics of parent-child relationships, this study delved into the prevalence and consequences of PABs experienced by children, as reported by those children as adults. Surveying a diverse pool of participants intended to be representative of the UK general population, the research uncovered a high prevalence of PABs experienced by children, as well as a significant correlation between exposure to PABs and adverse mental health outcomes in adulthood. This is the first study in the UK to examine the prevalence of PABs in children and their outcomes.

### Prevalence of PABs

The present study found that, using the most conservative categorizations, most of the sample had experienced at least one alienating behavior by mothers and fathers, with around a fifth experiencing more than 10. When using an expanded categorization (including sometimes), figures were significantly higher, with around a quarter of the sample experiencing over 20 alienating behaviors from mothers or fathers. This supports previous work by Verrocchio and colleagues (27) which found that around 58% of their adult sample had experienced some form of PABs by mothers, and 46% by fathers, at some point across childhood. Importantly, our data demonstrate that PABs are not without consequence, as they were significantly related to the acceptance and rejection of parents by exposed children. Our data, therefore, significantly advances prior studies, offering a robust UK-centric perspective. Specifically, these findings suggest that thousands of children are likely to experience PABs every year in the UK, and worldwide.

### PABs and mental health

Our findings also demonstrate a signficant correlation between experiencing PABs in childhood and adverse mental health outcomes, including PTSD symptoms, depression symptoms, and lifetime suicide ideation. This finding aligns with the substantial evidence base that details the profound effects of PA on children (12), and with specific studies examining the predictive impact of PABs on adolescent mental health (36, 37). Both the results from this and previous studies thus support the contention that PABs, by definition, are psychologically distressing and can have far-reaching consequences on mental health. We also found that although our sample composition was predominantly female and there was less power to detect gender differences in our analyses for males, males were more likely to have experienced PABs than women. This finding demonstrates that whilst mothers and fathers are equally capable of perpetrating PABs (22, 23), they may be more likely to do so toward sons; a finding that requires further investigation. For other demographic characteristics, our study found no significant differences in the experience of PABs, emphasizing that PA can affect a wide range of individuals.

### Implications

The findings of this study have important implications for legal, clinical, and intervention efforts. From a legal and policy perspective, the profound mental health impacts associated with PABs necessitate urgent and systematic action by UK policymakers and the legal system. These findings should act as a catalyst for the development of targeted interventions, the drafting of guidelines, and the creation of policies aimed at addressing and mitigating these behaviors. Indeed, it is positive that recent guidelines from the Family Justice Council in the UK have acknowledged PABs (labeled as simply alienating behaviors therein), and the impact they can have, as well as providing a framework for assessing claims brought to court. However, various elements of this guidance, including a fundamental acceptance of the phenomenon, need to be much stronger. Moreover, this guidance still promotes a distinction of severity and seriousness between PABs and other forms of domestic violence (classing the latter as more “serious”). Instead, these results underscore the need for comprehensive legal frameworks that prioritize the wellbeing of affected individuals and acknowledge PABs as a legitimate and hugely damaging form of family violence.

Clinically, mental health professionals must also be equipped with a deep understanding of the implications of PABs for the mental health of parents, particularly given its associations with severe conditions such as PTSD and depression. Enhanced training and awareness are essential to ensure that practitioners can identify and address the complex mental health needs of those impacted by PABs. Moreover, these findings highlight the critical importance of early intervention to prevent the normalization of PABs. Efforts should focus on challenging and reshaping narratives around acceptable behavior within both intact and separated family systems. Broader conversations about family dynamics and the processes surrounding separation must be fostered to address these behaviors proactively. Interventions at this systemic level are vital for promoting healthier relationships and reducing the prevalence of PABs, ultimately protecting the mental health of children and parents alike.

An important feature of the present findings is that they capture experiences of parental alienating behaviors (PABs) from the perspective of children, reported retrospectively by young adults. Previous prevalence studies have largely relied on reports from parents themselves [e.g., (22–24)]. While parent-report studies provide important insight into the experiences of targeted parents, the present findings demonstrate that children themselves also report widespread exposure to these behaviors and identify similar associations with relational and mental health outcomes. Taken together, these perspectives suggest that PABs can be observed across multiple vantage points within the family system, highlighting the importance of considering both parent and child reports when examining their prevalence and impacts.

### Limitations

This study, while providing important insights into the prevalence and impacts of PABs, is subject to several methodological limitations that warrant consideration, Firstly, the reliance on self-reported data introduces potential biases, as participants' responses may be influenced by memory inaccuracies or tendencies to present themselves in a favorable light. This issue is particularly pronounced when addressing sensitive topics like abusive behaviors. To mitigate these concerns, the study incorporated multiple measures and approaches, such as examining PABs through various lenses, including beliefs and behaviors. Additionally, the survey responses were collected anonymously, and validated measures with time-limited items (e.g., reporting behaviors over the past year) were used to minimize recall biases and self-presentation effects. We also sampled young adults no older than 25 years of age to minimize recall biases.

Another significant limitation is the study's cross-sectional design, which captures data at a single point in time and therefore does not allow for direct assessment of the directionality of the relationships observed. While this study identifies significant associations between exposure to parental alienating behaviors (PABs) and adverse mental health outcomes, it cannot determine causal ordering. However, longitudinal research has begun to address this limitation. For example, several studies using Chinese populations demonstrate prospective pathways from exposure to parental alienating behaviors to later mental health difficulties in adolescents (36–38). Taken together, these findings suggest that PABs are likely to function as a risk factor for subsequent mental health outcomes, though further longitudinal work is needed to fully understand these developmental trajectories.

A further limitation concerns the gender distribution of the sample, which contained proportionally fewer male than female respondents. Although the recruitment strategy was designed to approximate national representativeness using panel-based probability sampling, no strict gender quotas were imposed within the eligible age group. As such, the final gender composition reflects the natural response distribution within the recruitment panel rather than an intentional sampling restriction. Importantly, the statistical analyses employed in this study do not rely on equal group sizes and are robust to unequal sample distributions. Nevertheless, the gender imbalance should be considered when interpreting gender-specific findings, and future research would benefit from replicating these results using samples with a more balanced gender composition.

Methodological challenges inherent in working with adults reporting on their childhood experiences also present notable limitations. Adults reflecting on past behaviors may misinterpret or misattribute certain actions due to incomplete understanding or lack of broader context at the time. For instance, behaviors they perceived as alienating could have been protective measures implemented to shield them from an abusive parent. This possibility introduces the potential for significant bias and highlights the need for caution when interpreting self-reported data. Future research should account for these complexities by incorporating methods that triangulate self-reports with additional sources of information or contextual data, ensuring a more nuanced understanding of the motivations and circumstances surrounding such behaviors.

Finally, while coercively controlling abusive dynamics in families where the children are subjected to PABs can result in PA of the child, this outcome is not fated for every child (23). Indeed, some children are resilient to the coercive efforts of the alienating parent and researchers have started to identify protective factors to better understand why some children are more vulnerable than others [e.g., enforcement of parenting time with the targeted parent; (24)]. The purpose of the current study was to examine the extent and impact of PABs on children, as even the perpetration of one PAB is rated by most adults as being considered abusive toward children (8). Future research will need to examine how many children ultimately become alienated (aligned with the perpetrating parent *and* rejecting the targeted parent). As the measurement of PABs and PA evolves to reflect empirical and theoretical advances, future researchers will be able to more accurately understand the scope and impact of these dynamics on family members. Furthermore, the covert nature of PABs and the stigma surrounding these behaviors likely contribute to underreporting. This issue suggests that the true prevalence and impact of PABs may be higher than reported, emphasizing the need for methodologies that can better capture the full scope of these behaviors.

## Conclusion

The present study suggests that the prevalence and impact of PABs in the UK is alarmingly high and may significantly affect children's mental health into adulthood. Results specifically highlight the widespread nature of PABs, with most participants experiencing these behaviors, as well as a significant correlation to PTSD symptoms, depression symptoms, and suicidal thoughts. To address these problems, comprehensive legal reforms, educational initiatives, mental health support, and public awareness campaigns are urgently needed. These steps are crucial to protect children from the severe psychological harm caused by PABs and to foster healthier family dynamics.

## Data Availability

The raw data supporting the conclusions of this article will be made available by the authors, without undue reservation.

## Footnotes

1 Harman JJ, Warshak R, Sharples A, Marsden J. Parental alienation or blowback: rethinking the impact of parental denigration. *Manuscript under review*. (2024).

## Appendix A – Parental alienating behaviors (PABs)

Below is a list of the PABs measured in this study. The example below is asking about mothers' alienating behaviors.

1. It was best that I didn't tell my mother about fun things I did with my father when we spent time together.
2. My mother wanted to know if I liked her more than my father.
3. My mother has told me not to tell my father about when I have school or sport activities.
4. My mother and I made up stories about my father and we have told the stories to people like the police or the doctor so that I would not need to see him anymore.
5. My mother said my father was not responsible enough to make decisions for me.
6. My mother put down my father's values.
7. My mother has made a lot of sacrifices for me and so I needed to support her.
8. My mother called or texted to check up on me when I was with my father.
9. When my parents were in the same room, my mother spoke to father very respectfully in front of me.
10. My mother encouraged me to have a strong relationship with my father.
11. My mother's extended family has not had many nice things to say about my father.
12. My mother encouraged me to spend less time with my father.
13. My mother made me feel guilty if I enjoyed time with my father.
14. My mother and/or I blocked my father's phone number so that I would not have to talk to him.
15. I have told my mother things about what my father was doing in order to help her with a court case.
16. When talking about my father, my mother used a tone of voice that made me think that she had negative feelings about my father.
17. When my parents were in the same room, my mother talked down to my father in front of me.
18. My mother and I have been best friends since I was young.
19. I have shown my mother my text messages between me and my father.
20. My mother made me feel like I was free to give love to or receive love from my father.
21. My mother spoke positively about my father.
22. My mother's extended family let me know they did not think my father was a good parent.
23. My mother's extended family complained about my father.
24. My mother made comments to indicate that my relationship with my father was not important.
25. I feel like my mother tested me to make sure I was on her side.
26. My mother has waited outside when I have had to visit or see my father, just in case I felt unsafe.
27. My mother said bad things about my father in front of me.
28. My mother has told me that it is us against the world and that we need to stick together.
29. My mother wanted me to feel very close to both of my parents.
30. The extended family on my mother's side talk badly about my father.
